# The Global Maternal and Newborn Health Platform: study protocol for an observational, multi-country study on the quality of intrapartum and early postnatal care at health facilities

**DOI:** 10.1186/s12889-026-26468-4

**Published:** 2026-07-18

**Authors:** The Global Maternal and Newborn Health Platform – Asia-Pacific Research Group, Joshua P Vogel, Caroline SE Homer, Fiona Bruinsma, Delly Babona, Kara Blackburn, Skandarupan Jayaratnam, Long Nguyen, Naomi E Spotswood, Anita Voloshin, Mercedes Bonet, Tina Lavin, Khurshed Nosirov, Olufemi T Oladapo, Soe Soe Thwin, Minh D Pham, Salahuddin Ahmed, Sabbir Ahmed, Ahad Mahmud Khan, Nur-A-Safrina Rahman, Mohammod Shahidullah, Adama Baguiya, Mabel Berrueta, Veronica Pingray, Kitty Bloemenkamp, Emily Karahalios, Jermias Da Cruz, Harendra Dassanayake, Louise-Tina Day, Farhana Dewan, Kapila Jayaratne, Chandana Jayasundara, Athula Kaluarachchi, Robert Jones, Glen Mola, Tina Lavendar, Tippawan Liabsuetrakul, Nurlisa Oomudee, Pisake Lumbiganon, Glen Mola, Litia Narube, Detty Siti Nurdiati, Likke Prawidya Putri, Bayu Satria Wiratama, Leeanne Panisi, Nina Pio, Freda Pitakaka, William Pomat, Sandesh Poudel, Zenaida Dy Recidoro, Milena lay dos Santos, Lisa Vallely, Buyanjargal Yadamsuren, Khalid Yunis

**Affiliations:** 1 Global Women’s and Newborns’ Health Working Group, Burnet Institute, Melbourne, Australia; 2 Department of Sexual, Reproductive, Maternal, Child and Adolescent Health and Ageing, World Health Organization, Geneva, Switzerland; 3 Global Adolescent Health, Murdoch Children’s Research Institute, Melbourne, Australia; 4 Projahnmo Research Foundation, Dhaka, Bangladesh; 5 Institut de Recherche en Sciences de la Santé (IRSS), Ouagadougou, Burkina Faso; 6 Instituto De Efectividad Clinica Y Sanitaria (IECS), Buenos Aires, Argentina; 7 University Medical Center, Utrecht, Netherlands; 8 Melbourne School of Population and Global Health, University of Melbourne, Parkville, VIC Australia; 9 World Health Organisation, Timor Leste Country Office, Dili, Timor-Leste; 10 Ministry of Health, Colombo, Sri Lanka; 11 London School of Hygiene & Tropical Medicine (LSHTM), London, UK; 12 Obstetrical and Gynaecological Society of Bangladesh, Dhaka, Bangladesh; 13 Faculty of Medicine, University of Colombo, Colombo, Sri Lanka; 14 Port Moresby General Hospital, Port Moresby, Papua New Guinea; 15 Liverpool School of Tropical Medicine Liverpool, Liverpool, UK; 16 Prince of Songkla University, Hat Yai, Thailand; 17 Khon Kaen University, Khon Kaen, Thailand; 18 University of Papua New Guinea, Port Moresby, Papua New Guinea; 19 Fiji National University, Suva, Fiji; 20 Faculty of Medicine, Gadjah Mada University, Yogyakarta, Indonesia; 21 Department of Obstetrics and Gynaecology, National Referral Hospital, Honiara, Solomon Islands; 22 Aspen Lautoka Hospital, Lautoka, Fiji; 23 Ministry of Health and Medical Services, Honiara, Solomon Islands; 24 PNG Institute of Medical Research, Goroka, Papua New Guinea; 25 Paropakar Maternity and Women’s Hospital, Kathmandu, Nepal; 26 Independent Consultant, Manila, Philippines; 27 Guido Valadares National Hospital, Dili, Timor-Leste; 28 Kirby Institute, University of Sydney, Kensington, NSW Australia; 29 Obstetric Gynecology Professional Advisor Team, Ministry of Health, Ulaanbaatar, Mongolia; 30 American University of Beirut, Beirut, Lebanon

**Keywords:** Intrapartum, Maternal, Newborn, Postnatal, Quality of care

## Abstract

**Background:**

Good-quality care in the intrapartum and early postnatal period are critical to ensuring maternal, fetal and newborn survival and well-being. There is currently no standardised approach to assess the quality of intrapartum and early postnatal care provided in health facilities, women’s experiences of that care, and whether the services provided are aligned with the latest WHO recommendations. The Global Maternal and Newborn Health Platform (GMP) aims to establish and sustain a multi-country network of health facilities providing childbirth services, to measure and improve the quality of intrapartum and early postnatal care. The main objectives of this Platform are (i) to measure coverage of key intrapartum and early postnatal care practices and their alignment with WHO recommendations, (ii) to describe women’s experiences of care, (iii) to measure key maternal and newborn health outcomes.

**Methods:**

GMP will use periodic, cross-sectional, observational data collection in a multi-country network of health facilities from up to 63 low- and middle-income countries (LMICs). In the first wave, GMP is being implemented in 74 facilities in 10 Asia–Pacific countries. In participating facilities data will be collected at level of the individual woman/baby, maternal and newborn health providers, and facility.

**Discussion:**

This multi-country initiative aims to assess the quality of intrapartum and early postnatal care in health facilities, with a “person-centred” approach that emphasizes women’s experiences during birth and postnatal admission. In its first wave, data will be collected from over 100,000 women and their babies, alongside nearly 30,000 pre-discharge surveys on care experiences, complemented by responses from over 2,000 healthcare providers across 74 facilities in 10 Asia–Pacific countries. The resulting dataset will enable multi-country, country-specific, and facility-level analyses to identify actionable priorities for improving maternal and newborn health outcomes. GMP’s tools and methods are developed using an evidence-based approach and foster multidisciplinary networks among healthcare professionals, researchers, and policymakers. GMP provides a robust, scalable approach for periodic and standardised data collection, informing evidence-based policy and practice to enhance care quality. GMP will provide global situational analyses on intrapartum and early postnatal care quality to address the Sustainable Development Goals (SDG) and will help to addresses the WHA 77.5 resolution to accelerate progress towards reducing maternal, newborn and child mortality, including stillbirths.

**Supplementary Information:**

The online version contains supplementary material available at 10.1186/s12889-026-26468-4.

## Background

In the past three decades, the proportion of women attending health facilities to give birth and having access to skilled care has grown worldwide, while maternal and newborn mortality rates have decreased [1]. Yet, latest estimates of maternal and newborn mortality suggest progress has stagnated—around 287,000 maternal deaths, 1.9 million stillbirths and 2.3 million newborn deaths occur each year [2–4]. The vast majority of deaths are in low- and middle-income countries (LMICs), mainly in Africa and Asia, where many countries are not on track to meet the Sustainable Development Goals (SDGs) mortality targets [5]. In response to these realities, the World Health Assembly (WHA) passed in 2024 a resolution to accelerate progress towards reducing maternal, newborn and child mortality, including stillbirths [6].

High-quality care during labour, childbirth and the postnatal period is critical to ensuring maternal, fetal and newborn survival and well-being [7, 8]. The World Health Organization (WHO) quality of maternal and newborn care framework emphasises the need to optimise provision and experience of care, and ensure maternal and newborn health services have the appropriate resources to provide both (Supp. Figure 1). In line with these global agendas, WHO’s recommendations on intrapartum and postnatal care [9, 10] emphasise respectful, individualized, evidence-based, person-centred care to optimise health and wellbeing for the woman and her baby [11].

A long-standing challenge for maternal and newborn care quality in many LMICs is the lack of actionable, reliable data systems [12, 13]. Without such systems, the magnitude and causes of maternal and newborn mortality and morbidity, the coverage of key clinical and non-clinical interventions, the availability of resources, and whether these measures are improving over time cannot be tracked. Recent scoping reviews indicated that the measures used for intrapartum and early postnatal care monitoring are poorly aligned with current WHO recommendations for maternal and newborn care [12, 13]. A forthcoming systematic review found that, with the exception of Latin America, there are few sustained maternal and perinatal data systems in high-burden countries, and fewer still that align with WHO-recommended care practices (V Diaz, personal communication). Some LMIC-based research studies have comprehensively measured the quality of intrapartum care provision [14–16] however such systems are not harmonized for global implementation.

A second, related challenge is that routine approaches to maternal and perinatal data collection systems have not caught up with the broader shift towards person-centred, respectful care, and the importance of women and parent’s experiences [12]. The mistreatment of women and newborns around the time of birth is worryingly common [17, 18]. A more holistic approach is using person-centred data on women’s birth experiences to complement metrics on care provision and health outcomes. Together, these can produce a comprehensive evaluation of maternal and newborn care quality, and better direct quality improvement efforts. It is nonetheless complicated to implement at scale, requiring validated, reliable and culturally appropriate tools for measuring women’s care experiences [19].

A third challenge is that international maternal and newborn care recommendations evolve over time. WHO’s intrapartum and postnatal care guidelines have shifted significantly in the past several years in response to new evidence. For example, major changes to how labour should be monitored and managed, and stronger emphasis on the role of supportive care interventions during the intrapartum and postnatal period [20, 21]. While several countries have rapidly adopted latest WHO recommendations [22–24], it is not well known to what extent these have reached real-world clinical practice.

### Rationale for the Global Maternal and Newborn Health Platform

There is currently no comprehensive approach to evaluate the quality of intrapartum and early postnatal care against the latest WHO recommendations. Such an approach is needed to comprehensively evaluate the quality of care in facilities, monitor facility performance over time and prioritize interventions to improve quality of care, while incorporating measurement of provider practices as well as women’s experiences.

Over the past 15 years, WHO has led several multi-country observational studies on maternal and perinatal health outcomes [8, 25–27]. These studies generated new, practice-changing evidence related to mode of birth, maternal near-miss, and prevention and management of complications of abortion and maternal infections. They also provide a platform by which large-scale observational studies and research strengthening activities can be conducted.

The Global Maternal and Newborn Health Platform (GMP) builds on and extends these previous multi-country studies. The platform will conduct periodic analyses on intrapartum and early postnatal care quality, and associated maternal, fetal and early newborn health outcomes. It will help countries and health facilities assess their practices alongside WHO recommendations, and it will also provide opportunities to disseminate evidence-based guidance to facility and national level health stakeholders, accelerating improvements in quality of care.

### Focus on Asia–Pacific

The Asia–Pacific region with 36 LMICs (22 in Asia and 14 in the South Pacific) exemplifies these challenges—10 women in this region die every hour due to pregnancy-related causes [28, 29]. There is also a disproportionately high burden of severe maternal morbidity in health facilities in Asia–Pacific LMICs [30]. Women and families accessing health services in many Asia–Pacific countries face numerous challenges including poor-quality antenatal, childbirth and postnatal care, significant shortages in the health workforce, and a lack of data on facility-based maternity care quality, coverage of essential interventions, and health outcomes [28, 29]. Furthermore, the Asia–Pacific region, especially the smaller countries, are rarely included in global research efforts to improve maternal and newborn health.

### Aim and objectives

The overall aim of GMP is to establish and sustain a multi-country network of health facilities providing childbirth services, to measure and support improvement in the quality of intrapartum, and early postnatal care.

Primary objectives:

1. Measure coverage of key intrapartum and early postnatal care practices in participating health facilities and whether key WHO intrapartum and early postnatal care recommendations are being implemented;
2. Measure women’s experience of intrapartum, and early postnatal care at participating health facilities;
3. Measure frequency of key maternal and newborn health outcomes related to the intrapartum and immediate postnatal period;

Secondary objectives:

4. Support dissemination and uptake of WHO guidelines and associated tools related to intrapartum and early postnatal care in health facilities;
5. Facilitate data-driven approaches to improving quality of intrapartum, and early postnatal care in participating health facilities;
6. Strengthen maternal and newborn health research capacity in participating countries and health facilities.

## Methods

### Study design

**Fig. 1:**
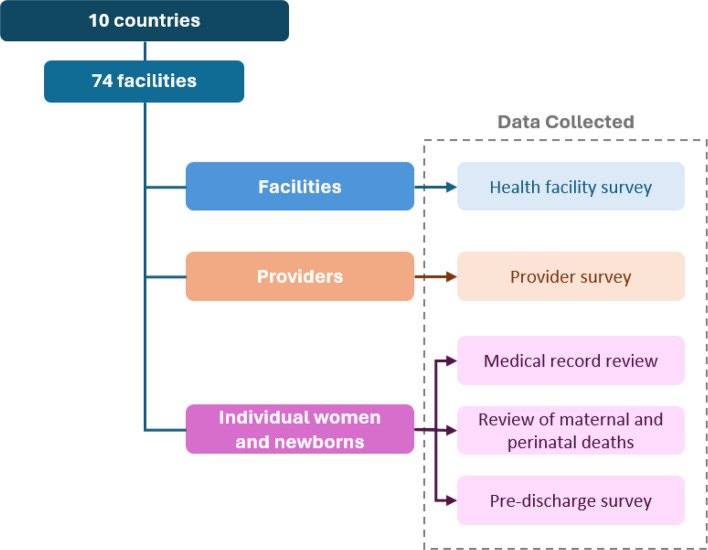
Overview of GMP Study design and activities

**Fig. 2:**
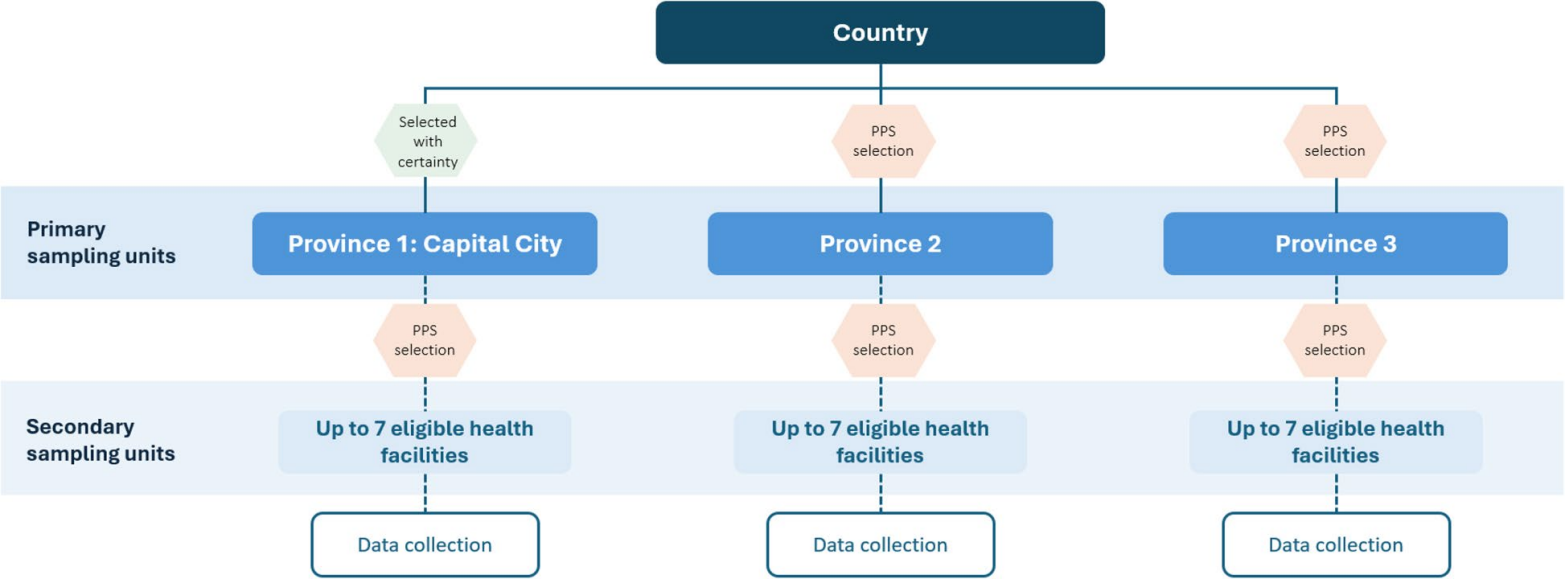
Overview of sampling strategy Note: PPS = proportional probability sampling

GMP will use a prospective, facility-based, observational design. It collects data on intrapartum and postnatal care practices, the experiences of women giving birth, and their health outcomes (Fig. 1). It employs a multi-stage sampling strategy similar to that used in previous WHO multi-country surveys of maternal and newborn health [8, 25, 31] (Fig. 2). It is expected that this core GMP protocol will ultimately be implemented in over 1,000 health facilities in up to 63 LMICs, across six WHO regions (Supplementary Table 1). Selection of countries and health facilities, and data collection will follow a similar approach across all regions.

#### Selection of countries

We pre-identified 17 countries in the South-East Asia (SEA) and Western Pacific (WP) WHO regions, hereafter referred to as the ‘Asia–Pacific’, (Table 1) that could potentially participate in the first round of the GMP. The initial list was informed by countries participating in previous WHO multi-country surveys, and consultations with WHO headquarters, WHO regional and country offices, and national Ministries of Health. This process aimed to ensure diversity in terms of burden of maternal and perinatal health outcomes, perceived quality of maternal and newborn health services, alignment with regional priorities and initiatives, leveraging existing research networks and previous research capacity investments, willingness of the country to participate, and financial costs and feasibility. It will also allow assessment of some maternal and perinatal indicators over time, through comparison with data from previous surveys. In the first phase of GMP in the Asia–Pacific, 10 countries will participate: Bangladesh, Nepal, Sri Lanka, Thailand, and Timor-Leste in the SEA region; Fiji, Indonesia, Mongolia, Papua New Guinea, Solomon Islands in the WP region. Four rounds of data collection at 3-year intervals (2024, 2027, 2030, 2033) are planned to enable monitoring trends of quality of care at participating health facilities over time. It is expected the same countries will participate in subsequent rounds in the Asia–Pacific.

**Table 1:** List of countries considered for participation in the Global Maternal and Newborn Health Platform (GMP) Asia–Pacific

South-East Asia region	Western Pacific Region region
1. **Bangladesh**	1. Cambodia*
2. India*	2. China*
3. Nepal*	3. **Indonesia**
4. Sri Lanka*	4. Mongolia*
5. Thailand*†	5. Philippines*
6. **Timor-Leste**	6. Viet Nam*†
	7. **Fiji**
	8. Laos
	9. **Papua New Guinea**
	10. Samoa
	11. **Solomon Islands**

^*^Participated in previous WHO multi-country surveys (WHO Global Survey [25], WHO Multi-country Survey [32] and/or WHO Global Maternal Sepsis Study (GLOSS) [27]

†HRP Alliance hubs are located in these countries

Countries participating in GMP Asia–Pacific are shown in bold

#### Selection of provinces/states

From each participating country, three provinces/states are selected: the province/state where the country’s capital city is located, and two randomly sampled provinces/states (with probability proportional to the population size). In situations where one of the selected provinces/states has too few facilities (i.e. only one or two eligible facilities) a fourth province/state is selected (as described below). To be eligible, provinces/states needed to have an institutional birth coverage of at least 30%, and at least one eligible facility (Fig. 2).

#### Selection of facilities

Within each sampled province/state, up to 7 eligible facilities are randomly sampled, with a probability proportional to births per year. Eligible facilities are those with a minimum of 2,000 births per year, the capacity to perform Caesarean section and can be of any type (public, private or other). If less than 7 eligible facilities are in the province/state, all facilities are selected. Where there are only 1 or 2 eligible facilities in a province/state, an additional (fourth) province/state is selected. Facility networks in countries that participated in previous WHO surveys [8, 25, 32] will be re-activated wherever possible [32]. However, some of these facilities may no longer meet the eligibility criteria, or may decline participation. In such situations new facilities from the same province/state are sampled using the aforementioned sampling strategy (Fig. 2).

#### Data collection period

All participating health facilities will collect data for a minimum of 3 months. If a sample size of 1,000 births per facility cannot be reached by 3 months, data collection will continue to a maximum of 6 months.

### Study populations

GMP will collect data at three levels: individual woman/baby level, provider level and facility level.

#### Individual woman/baby level

Data is collected during the women’s admission for birth until discharge, death, transfer or it is 7 days after birth, whichever comes first. All women giving birth at 22 weeks’ gestation (or if gestation is unknown, 500 g birthweight) in the participating health facilities during the study period will comprise the study population, regardless of their maternal and perinatal outcomes. Women who give birth outside the study health facility, or pregnant women who are admitted but do not give birth, are excluded. If any maternal or newborn deaths or stillbirths are identified, further information on cause of death is collected. A random sample of women will be invited to complete a pre-discharge interview (Fig. 1).

#### Provider level

The study population are any skilled health workers employed at the participating health facility and who provide intrapartum or postnatal care during the study period. These may be doctors, nurses or midwives at any level of seniority. Students, such as midwifery, nursing or medical students, are not included (Fig. 1).

#### Facility level

One facility survey will be completed per facility (Fig. 1). Information will be obtained through interviewing the Head of Department of Obstetrics and Gynaecology, and the Head of the Department of Neonatology/Paediatrics and/or the Head of Nursing and Midwifery (or their representatives), as well as facility managers or relevant department staff (e.g. pharmacy).

### Primary and secondary outcomes

GMP has 18 primary (Table 2) and 29 secondary outcomes (Tables 3 and 4) spanning the intrapartum and early postnatal periods. These outcomes include coverage of key practices, people-centred care (experience) outcomes and health outcomes, for women and newborns. Operational definitions for all outcomes are presented in Supplementary Table 2.

**Table 2:** Primary outcomes for the Global Maternal and Newborn Health Platform

	**Woman**	**Newborn**
**Coverage of key practices** (Medical record)	• Labour monitored using partograph* • Prophylactic uterotonic administered immediately after birth* • Pre-discharge counselling provided to woman prior to discharge*	• Breastfeeding initiation < 1 h after birth*
**Woman-centred outcomes** (Pre-discharge survey)	• Companion of choice during labour and birth * • Any experience of mistreatment during time in the health facility for childbirth* • Effective communication – consent obtained for vaginal examinations • Satisfaction with care	• Skin to skin contact during the first hour after birth*
**Health outcomes** (Medical record)	• Caesarean section (Robson and overall)* • Episiotomy • 3rd/4th perineal tear* • Hysterectomy	• Stillbirth (antepartum and intrapartum • Early neonatal mortality • Preterm birth < 37 weeks • Low birthweight < 2500 g • Apgar score less than 7 at 5 min

^*^Providers and facilities will also be asked about policy and practice relating to these outcomes

**Table 3:** Secondary outcomes – intrapartum period

Type	Specific outcome	Source
**Coverage of key practices**	Amniotomy (overall, early amniotomy)	Medical record
	Oxytocin for augmentation	Medical record
	Operative vaginal birth	Medical record
	Routine antibiotic prophylaxis before birth	Medical record; provider survey
	Fundal pressure	Provider survey
	Routine use of cardiotocography	Provider survey
**People-centred outcomes**	Use of pain relief during labour/birth and after birth	Medical record survey; Pre-discharge women’s survey; Provider survey
	Oral food/fluid intake	Provider survey
	Birth position	Provider survey
	Mobilising during labour	Pre-discharge women’s survey; Provider survey
**Health outcomes**	Maternal death occurring during admission for childbirth, up to 7 days postpartum	Medical record
	Maternal ICU admission for > 24 h	Medical record
	Postpartum haemorrhage (PPH) requiring use of additional uterotonic/TXA, uterine balloon tamponade or surgical intervention (excluding hysterectomy), blood transfusion	Medical record
	Intrapartum related perinatal mortality: intrapartum stillbirth + very early neonatal death (in first 24 h after birth)	Medical record
	NICU admission > 24 h	Medical record

**Table 4:** Secondary outcomes – postnatal period

Type	Specific outcome	Source
**Coverage of key practice**	Intramuscular vit K injection of newborn administered	Medical record
	Newborn bathing delayed until 24 h after birth	Provider survey
	Screening for neonatal hyperbilirubinemia (using TcB)	Medical record
	Newborn immunization with Hep B vaccine	Medical record
**People-centred outcomes**	Pharmacological pain relief offered to woman	Pre-discharge survey; Provider survey
	Pre-discharge counselling provided to woman prior to discharge	Medical record, pre-discharge survey
	Postnatal contraceptive information provided to women	Pre-discharge women’s survey; provider survey
	Newborns treated with respect/mistreated	Pre-discharge survey
	Women separated from newborn for non-medical reasons	Pre-discharge survey
**Maternity care**	Midwifery continuity of care	Facility survey
	Policies around clinical interventions (e.g. episiotomy, routine amniotomy)	Facility survey
	Cleanliness and sanitation	Facility survey
	Availability of power/water	Facility survey
	Availability of essential supplies (including medicines)	Facility survey

### Sample sizes

GMP will collect individual-level data on multiple outcomes, for which the prevalence may vary. For example, in the WHOMCS early breastfeeding rates ranged from 17.7% to 98.4% [33]. Some adverse health outcomes (e.g. stillbirth) may be less frequent in low-burden facilities or countries. We explored different sample size scenarios for a range of GMP outcomes and levels of precision. For example, for a relatively rare outcome (e.g. 0.7% stillbirth rate) at ± 0.5% precision, 1068 women are required. For a more frequent outcome (e.g. 12% Caesarean rate) and ± 2.0% precision, 1,015 women are required. We also noted that in the WHOMCS, the average number of women per facility was 876. Through consultations with the study country investigators and advisory group and investigators, we opted for a pragmatic target of at least 1,000 women per facility.

For the pre-discharge women’s survey, we estimate that ~ 80% of women giving birth will be eligible. For a given outcome on women’s birth experiences (hypothetical prevalence ranging from 5 to 95%) and ± 5% precision, 385 women are needed. We therefore will sample a minimum of 385 women per facility.

For the provider survey, acknowledging that survey response rates may be less than 50%, we aim to maximise participation and representativeness. Hence, all staff providing intrapartum, and postnatal care at GMP facilities are eligible, and will be approached invited to participate.

### Data collection instruments

We sought a measurement approach that would be relatively low-cost and feasible to implement in limited-resource settings. First, we mapped all WHO intrapartum and early postnatal care recommendations and best practices, identifying where and how each of these could be measured (e.g. woman/baby, provider or health facility level). For example, the broad concept of pain relief during labour and birth could be identified from a medical record, by asking a woman whether she was offered pain relief, asking a provider if they routinely offer pain relief, or assessing whether a facility has pain relief options available.

We considered this mapping and prioritised what to measure based on 1) clinical importance, 2) prevalence or coverage of the recommendation, 3) relative importance of that recommendation to quality of maternal and newborn care more broadly, 4) measurability, 5) balance of benefit and harm within evidence supporting the recommendation. We also ensured GMP’s primary and secondary outcomes were captured, which also evolved alongside this process. This was an iterative process, including literature reviews and expert consultations. We also reviewed existing instruments which had been used in previous multi-country studies to measure aspects quality of maternal and newborn care, identified from our scoping reviews [12, 13]. This resulted in the scope, content and first iterations of five GMP instruments. There are three instruments at the individual woman/baby level: 1) a medical record review form (for all women giving birth and their newborns), 2) a cause-of-death form (for any maternal, fetal or early neonatal death), and 3) a pre-discharge women’s experience survey (for randomly sampled postnatal women). GMP also measures at: 4) the provider level (provider survey) and 5) facility level (facility survey). All instruments were drafted, reviewed by GMP country investigators and advisory group members, field-tested in GMP sites, translated (where required) and refined prior to study implementation. We developed study manuals for each instrument—all investigators, facility co-ordinators and data collectors will undergo standardised training prior to data collection. Details for each instrument are provided below.

1. *Medical Record Review Form:* Items included in the medical record review form were developed based on the iterative process outlined above. GMP study data are abstracted from medical records into a digital study form, around the time of discharge. Data are non-identifiable and include maternal characteristics, maternal risk factors, coverage of essential intrapartum and early postnatal interventions, mode of birth, and maternal, perinatal and newborn health outcomes. Facility medical staff can be approached if there are doubts or clarifications are required.
2. *Cause of death form:* If any maternal death, stillbirth or early neonatal death is identified, data on cause of death will be collected using a dedicated form using the WHO ICD-MM [34] and ICD-PM [35] classifications.
3. *Pre-discharge women’s experience survey:* The aim of this instrument is to understand the women’s perspective on the characteristics of good-quality care. Our ideal instrument would have good validity (particularly face and content validity), reproducibility and reliability, and would be feasible to use in limited-resource settings. From the literature reviews we identified four tools [36–38] with the greatest alignment to GMP’s objectives and needs. We extracted specific items from these tools and mapped them to our prioritised list. We developed a first iteration (version 1) using items adopted or modified from these tools, created new items where required, and organised this into a logical workflow. This was revised following consultations with WHO staff and three independent experts working in measuring women’s experiences after birth (version 2) and finalized by the research group (version 2.1) based on face and content validity, feasibility and understandability. We pilot-tested version 2.1 on paper with four to six postnatal women (vaginal and caesarean birth) per each GMP country. This was done by the principal investigator in each country using a cognitive interview guide, resulting in further revisions (version 2.2). A a digital audio computer-assisted self-interviewing (ACASI) survey was then created (Viewpoint, UK - https://viewpointorg.com/) for use on a tablet device [39]. The participant listens to a recorded voice (in a language of their choosing) and responds to questions via a touchscreen. Pictures are used for responses, as well as visual cues. This means women can participate regardless of literacy or language; headphones help ensure privacy. We pilot-tested tablet-based ACASI in one GMP site with four women, which indicated it was acceptable and feasible. For the Asia–Pacific region we translated the survey into 14 languages; each version was reviewed by investigators and bilingual speakers, back-translated and refined.
4. *Provider survey:* The aim of this instrument is to provide a standardized tool for assessing self-reported intrapartum and early postnatal care practices of healthcare workers and the factors influencing the implementation of these practices. A total of 12 validated, reliable and relevant questionnaires were identified from a rapid review of the literature, and 164 items were extracted and evaluated, mapped against WHO recommendations and prioritized by key concepts and domains based on a pre-defined conceptual model. A first version of the survey (version 1) was revised through cognitive interviews, conducted by an experienced social scientist in October to November 2023, with nine skilled health providers from two countries (Thailand and Papua New Guinea) to assess the survey's clarity, comprehensibility, and response capacity. Four healthcare providers from a GMP facility in Fiji completed the revised survey (version 2) and participated in group interviews to further assess the instrument's clarity, relevance, length and scale structure. The survey was finalized by the research group, pilot-tested in an electronic format, translated into eight languages for the Asia–Pacific, reviewed by investigators and bilingual speakers, and refined through back-translation (version 2.1).
5. *Health facility survey:* The facility survey captures data on the environment and context where intrapartum and early postnatal care is provided. It includes facility, volume and activity; organization and services (e.g. infrastructure, supplies, equipment, and human resources); and clinical policies and protocols for intrapartum and postnatal care. Following the recommendation mapping process described above, we reviewed WHO normative documents related to measuring intrapartum and early postnatal care at a facility level. This included WHO’s standards and monitoring framework for maternal and newborn care quality [40–42], WHO’s service availability and readiness assessment (SARA) tool [43], Harmonized Health Facility Assessment (HHFA) [44]; Service Provision Assessments (SPA) [45]; health facility surveys used in prior WHO multi-country studies [16, 25–27, 32] and a review of large-scale facility assessment tools to measure quality of maternal and newborn care as per the WHO framework [43, 46]. We extracted and consolidated specific, validated questions from these to form the first iteration of the facility survey (version 1). This was revised following consultations with GMP advisory members and measurement experts (version 2), field-testing in one GMP site, revised (version 3) and finalized in consultation with country teams (version 4).

### Data collection

Research staff are trained according to a standardized manual of operations, which minimizes the need for judgement and interpretation. Training workshops at country and facility levels are held prior to commencement of data collection. All collected data are stored on an access-restricted database. Participant confidentiality and anonymity will be maintained at all times by the sponsor, regional and country coordinators, and research staff in participating facilities. Processes for specific levels of data collection are described below.

#### Individual level data

Trained data collectors are deployed to participating facilities to collect GMP study data using tablet devices. Each day, data collectors review medical records or registers in labour and postnatal wards, as well as other clinical areas where birthing women may be present (such as emergency rooms or operating theatres), to extract individual level data. The total number of women admitted and giving birth at the facility each day is monitored using a customised log. Individual electronic study records are compared to logs to ensure eligible women are not missed.

The data system randomly selects women who have given birth to be invited to complete a pre-discharge survey. The sampling probability is based on the total number of expected births in that health facility and aims to ensure approximately 385 women are surveyed during a facility’s study period. Eligible women are those able to give informed consent and complete it (i.e. are not experiencing a serious adverse outcome). The data collector explains the survey to the woman using a participant information sheet in the woman’s language of preference. Informed consent is provided by agreeing to the consent statement on the tablet device. Survey completion occurs in a private environment within the health facility, on the tablet using headphones (if preferred).

#### Provider level data

The facility co-ordinator identifies all relevant skilled health workers (obstetrics, nursing, midwifery, neonatology, paediatrics) currently working at the facility in intrapartum and early postnatal care services. Providers are sent a unique link to complete the voluntary survey via email or mobile (WhatsApp or Line), or they can complete it using a study tablet. This survey obtains consent to participate, and captures data on their characteristics, as well as knowledge, attitudes and practices on intrapartum and early postnatal care.

#### Facility level data

A digital form is used to collect health facility level information. This is completed by the facility co-ordinator collecting the required information from the Head of Obstetrics & Neonatology/Paediatrics Departments (or their authorised representative) in the health facility. Some questions require input from different staff or departments (heads of unit, laboratory, infection prevention and control unit, administrative services).

### Data management

All data are collected electronically using tablets into Good Clinical Practice (GCP) compliant databases. Unique, pre-defined numbers are used for each participant. Where needed, study forms and manuals will be translated into relevant local languages. Data collection and entry procedures, storage, protection, ownership, sharing and retention will be compliant with the WHO standard operating procedures and GCP guidelines.

The REDCap database system (https://project-redcap.org/) is used to capture data for both the medical record review and the provider survey. REDCap uses data accuracy checks to minimize entry errors, facilitate monitoring and enable quick resolution of queries and missing data. For the medical record review form, a 10% random sample for the first 250 participants will undergo double data collection, with data compared for inconsistencies, and thereafter a 5% random sample of participants. Completed forms will be checked by facility, country and regional coordinators to ensure completeness, reliability and consistency of collected data.

Data curation and validity cross-checks will be performed within the centralised database. In-person monitoring visits by regional and country investigators will be organised during and after the data collection period, to evaluate protocol adherence and perform data quality verification. Additional visits will be carried out depending upon facility or country activity and performance. Upon completion of the study and verification of data for accuracy and completeness, the database will be locked from any additional changes.

Pre-discharge surveys will be prepared using ACASI in recordings of local languages. Data collected using the ACASI will be stored electronically in a central location associated with the woman’s unique identifying number and will not be available to the study site.

### Study governance

The WHO's Department of Sexual, Reproductive, Maternal, Child and Adolescent Health and Ageing is the global sponsor and is responsible for coordinating GMP in multiple WHO regions. In each region, there will be a Regional Coordinating institution. For Asia–Pacific countries this is the Burnet Institute, Australia. GMP is governed by a Steering Committee which includes the global sponsor (WHO-Geneva), regional coordinating institutions (e.g. Burnet Institute for the Asia–Pacific), independent advisors, and Country Principal Investigators. The Regional Co-ordinating Committee for the Asia–Pacific is responsible for overseeing implementation in this region, and comprises WHO-Geneva, Burnet Institute and Principal Investigators from the 10 participating Asia–Pacific countries. Each facility has a designated Facility Co-ordinator who will oversee all facility-level activities, and report to the Country Investigator.

### Ethics approval

The study was approved by The Alfred Hospital Human Ethics Committee (HREC for Burnet Institute) on 27 July 2023 (approval number 96879, 340/23), and the World Health Organization Ethical Review Committee (ERC.0004050) on 5 February 2024. This study will obtain all required authorizations at country and institutional level, and the relevant consent to participate.

#### Planned analyses

A statistical analysis plan (SAP) will be developed in collaboration with country researchers, study statistician and WHO statisticians, and finalised prior to completion of recruitment. Primary objectives 1–3 will use descriptive statistics at the individual, facility, or country level. Individual, facility and country level characteristics will be summarised using n (%) or mean (Standard deviation, SD) as appropriate. We will also explore possible associations between the use of interventions and women and newborn outcomes.

## Discussion

This ambitious, multi-country initiative aims to systematically assess the quality of intrapartum, and early postnatal care in health facilities across six WHO regions. A key novelty is GMP’s “person-centred” approach, collecting data on women’s experiences of care during birth and early postnatal admission. In the first wave of GMP in 74 health facilities in 10 Asia–Pacific countries, we anticipate collecting data from more than 100,000 women and their babies, as well as nearly 30,000 pre-discharge surveys on women’s experiences. This will be complemented by survey data from an estimated 2,000 providers or more working in these facilities. Collectively, the GMP model produces a world-first, comprehensive picture of intrapartum, and early postnatal care quality, inclusive of women’s and provider’s perspectives. This enables pooled analyses of multi-country data, as well as country- and facility-specific analyses to improve the quality of care.

The GMP network will create new opportunities for nested sub-studies, or additional research projects (alongside or between rounds of data collection). In the Asia–Pacific, GMP is catalysed by Burnet’s Centre for Research Excellence on Maternal and Perinatal Health in the Asia–Pacific (CRE-ARPAN), the HRP Alliance (WHO-Switzerland) and its hubs located in Thailand and Viet Nam. The GMP Research Group is actively seeking further funding to address further maternal and newborn health priorities and continue strengthening research capacity.

GMP’s intended impact is to improve the quality of intrapartum, and early postnatal care in health facilities. It will allow a standardised investigation of links between clinical environments and practices, women’s experiences, and maternal and perinatal outcomes. This enables a data-driven approach to setting priorities and taking action to improve maternal and newborn health. Results will be used for dialogue with healthcare providers, facility leadership, and state/national policymakers, and to target quality improvement initiatives. It will provide key data to guide policy and practice at a facility, provincial, country and regional level in the coming years. More broadly, GMP provides a validated, feasible approach to assess whether quality of intrapartum, and early postnatal care is consistent with current WHO evidence-based recommendations. GMP’s tools and methods will be made freely available for future use. GMP will help create and reinforce multidisciplinary networks between obstetricians, neonatologists, nurses, researchers, administrators, governments in participating facilities, countries and regions. Our ambition is that GMP data collection will be undertaken periodically (i.e. every 3 years).

### Strengths and limitations

A major strength of GMP is that it provides a real-world picture of intrapartum and early postnatal care quality in participating facilities. In addition, it expands and strengthens research networks from previous WHO-led multi-country surveys on maternal and newborn health [8, 25]. Expertise across partners spans research, maternal and neonatal clinical care, epidemiology and health policy. The large sample size, geographic and health system diversity enhances the applicability of results in the Asia–Pacific region and in future regions as they come on-board.

This study has some important limitations. In the facility selection criteria, including only those with at least 2,000 births per year means that this sample will be composed of mostly medium-to-large facilities, and many of which are tertiary referral hospitals. For relatively small countries (such as Solomon Islands, Fiji or Timor-Leste) this means only one or two health facilities are eligible. However, the inclusion of smaller countries, for the first time, in a WHO-led multi-country survey brings valuable diversity and generalisability. We anticipate that GMP’s tools and outputs will nonetheless be useful to lower-level or smaller facilities, though further investigation may be warranted. As GMP was not designed to cover home births the data cannot be generalised to births outside of health facilities.

## Conclusion

The data generated from GMP will provide a comprehensive picture of the quality of intrapartum, and early postnatal care at a range of health facilities in low- and middle-income countries. The first wave of 74 facilities in 10 countries of the Asia–Pacific will demonstrate this approach is feasible, insightful and scalable. It will generate baseline data for monitoring trends in the coverage of key WHO recommendations, women’s experiences, provider practices and maternal, newborn health outcomes – these can be periodically assessed over time. Bringing women’s experiences into measurement of quality of care on such a large platform is an important step forward to holistic, woman-centred quality of care measurement and improvement. GMP will demonstrate the importance of collecting and use of high-quality data from both provider and user perspectives for research, clinical and policy purposes. We hope that this network of health facilities and methodology will serve to help drive improvements in quality of facility-based intrapartum, early postnatal and care in LMICs across the region and eventually the globe.

## Supplementary Information

Supplementary Material 1

## Data Availability

No data were generated during the current status of the study. Data generated in the future will be subject to World Health Organization rules related to data sharing. Data ownership and use, and authorship of publications generated from the GMP Asia–Pacific project is governed by a GMP Data Use and Authorship Policy. Once the study is finalized and the results are published, a specific procedure for obtaining access to the database from WHO will be made publicly available. Study instruments in multiple languages are available on request from the authors.
