# Supplementary material for: The Global Maternal and Newborn Health Platform: study protocol for an observational, multi-country study on the quality of intrapartum and early postnatal care at health facilities
Source: BMC Public Health. 2026 Jul 18;26:2216. doi: 10.1186/s12889-026-26468-4 (PMC13397812; doi:10.1186/s12889-026-26468-4)
Supplement: Supplementary file 1 — Supplementary Material 1 [file 12889_2026_26468_MOESM1_ESM.docx]

**Supplementary Tables and Figures**


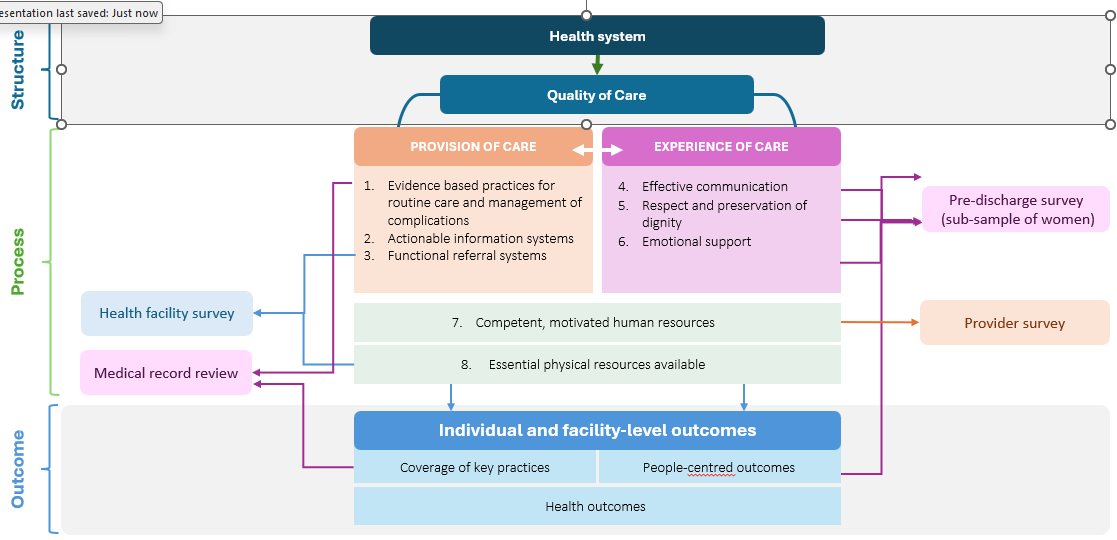


Figure S1. Diagram showing the different components of the WHO Quality of Care Framework for Maternal and Newborn Health (adapted from *Tuncalp et al. Quality of care for pregnant women and newborns the WHO vision. BJOG 2015 Jul: 122(8):1045-9).*

Table S1. List of countries that will be considered for participation in the Global Maternal and Newborn Health Platform (GMP)

| **African region** | **Americas region** | **South-East Asia region** | **Western Pacific Region region** | **Eastern Mediterranean region** | **European region** |
| --- | --- | --- | --- | --- | --- |
| 1. Benin‡ 2. Burkina Faso‡^a^ 3. Cameroon‡ 4. Cape Verde 5. Ethiopia†‡ 6. Ghana‡ ^§^ 7. Kenya* ^§^ 8. Malawi‡ 9. Mali‡ 10. Mozambique‡ 11. Niger* 12. Nigeria* 13. Senegal‡ 14. South Africa‡ 15. Tanzania 16. Uganda* 17. Zimbabwe‡ | 1. Argentina* 2. Bolivia‡ 3. Brazil* ^§a^ 4. Colombia‡ 5. Chile 6. Cuba* 7. Ecuador* 8. Guatemala‡ 9. Honduras‡ 10. Mexico* 11. Nicaragua* 12. Paraguay* 13. Peru* 14. Uruguay‡ | 1. **Bangladesh** 2. India* 3. **Indonesia** 4. **Nepal*** 5. **Sri Lanka*** 6. **Thailand***^§^ 7. **Timor-Leste** | 1. Cambodia* 2. China* 3. **Mongolia*** 4. Philippines* 5. Viet Nam*^§^ 6. **Fiji** 7. Laos 8. **Papua New Guinea** 9. Samoa 10. **Solomon Islands** | 1. Afghanistan* 2. Egypt†‡ 3. Jordan* 4. Lebanon* 5. Morocco†‡ 6. Pakistan* ^§^ 7. Palestinian Occupied Territory* 8. Sudan‡ | 1. Kazakhstan†‡ 2. Kyrgyzstan‡ 3. Lithuania‡ 4. Moldova‡ 5. Romania†‡ 6. Slovakia‡ 7. Tajikistan†‡ |

*Participated in previous WHO multi-country surveys (WHO Global Survey (25), WHO Multi-country Survey (32) and/or WHO Global Maternal Sepsis Study (27)

† Selected in Global Survey (REF) but did not participate

‡Participated in GLOSS (REF)

^§^ HRP Alliance hubs are located in these countries

Countries participating in GMP Asia-Pacific are shown in bold

Table S2. Operational definitions of study outcomes

| **Individual** | **Type** | **Specific outcome** | **Where measured** | **Denominator** | **Numerator** |
| --- | --- | --- | --- | --- | --- |
| Woman | Coverage of key practices | **Labour monitored using a partograph**    Outcome measures:    - Proportion of women who had their labour monitored using a partograph    - Type and % of each labour monitoring tool used    - Proportion of provider report using a partograph to monitor labour | *Medical record:*  Q. Was the labour monitored using a partograph = Yes    Q. If yes, what type = WHO simplified, WHO LCG, Other | All women who experienced labour (excludes no labour)      Total occurrence (instance) of partograph reported being use | Number of women who had their labour monitored using a partograph    Occurrence (instance) of each partograph reported being use |
|  |  |  | *Provider survey:*  Q25. Do you monitor the progress of a woman’s labour using a partograph or similar tool? = Yes | All providers who provide care during labour and birth | Number of provider reports use of labour monitoring tool (and type of tool) to monitor labour |
|  |  | **Prophylactic uterotonic administered immediately after birth**    Measure:  - Proportion of women who received prophylactic uterotonic immediately after birth | *Medical record:*  Q. Was prophylactic uterotonic given immediately after the birth of the baby? | All women giving birth | Number of women who received uterotonic immediately after birth |
|  |  | **Pre-discharge counselling provided to woman prior to discharge**    Measure:  - Proportion of women who received pre-discharge counselling on each/any/all topics | *Medical record:*    Q. Did the women receive pre-discharge counselling on any of the following topics (Breast feeding, Care of herself, postpartum danger signs and when to seek help, Care of the baby, Newborn danger signs and when to seek help, Family planning method)? = Yes | All women who gave birth and were discharged from hospital alive | Number of women who received pre-discharge counselling on (Each/Any/All) topic |
|  | Women-centred outcomes | **Companion of choice during labour and birth**    Measures:  - Proportion of women who wanted and had a companion during labour      - Proportion of women who wanted and had a companion during birth                        - Proportion of provider who reported that women can have a companion present during labour      - Proportion of provider who reported that women can have a companion present during vaginal birth    - Proportion of provider who reported that women can have a companion present during CS      - Proportion of provider who reported that the health facility has infrastructure to offer companionship | *Pre-discharge women’s survey:*    Q. During labour (before the baby was born), did you want to have a companion in the room to support you (such as a family member or friend)?    Q. Did you have a companion you wanted with you during labour (such as a family member or friend)?    Q. During the birth (when the baby came out), did you want to have a companion in the room to support you (such as a family member or friend)?    Q. Did you have a companion you wanted with you during the birth (such as a family member or friend)? | All women consented to and completed pre-discharge survey     - Who wanted labour companion;      - Who wanted birth companion | - Number of women who had a companion present during labour;    - Number of women who had a companion present during birth |
|  |  |  | *Provider survey: Answer for following Questions = Yes (most of the time/always)*    Q. Are women able to have a companion of their choice (any person chosen by the woman) to provide her with continuous support during labour, for as long as they want?    Q. Are women able to have a companion of their choice (any person chosen by the woman) to provide her with continuous support during vaginal birth, for as long as they want?    Q. Are women able to have a companion of their choice (any person chosen by the woman) to provide her with continuous support during caesarean section, for as long as they want?    Q. This unit has adequate infrastructure, equipment and supplies to offer companionship during labour and childbirth | All providers providing care during labour and birth | Offer women the option of having a companion present during labour/birth      - Number of providers who reported that women are able to have companion during labour    - Number of providers who reported that women are able to have companion during vaginal birth    - Number of providers who reported that women are able to have companion during CS    - Number of providers who reported that the health facility has infrastructure to offer companionship |
|  |  | **Experience of mistreatment**    - Proportion of women reported experiencing physical abuse    - Proportion of women reported experiencing verbal abuse    - Proportion of women reported experiencing stigma and discrimination    - Proportion of women reported any experience of mistreatment | *Pre-discharge women’s survey:*      Q. At any time during your time in hospital, did any of the following occur? = Yes   - You were pinched - You were slapped - Your were physically restrained     Q. During your time in the hospital, did any of the following events occur? = Yes   - You were shouted or screamed at by a hospital staff member - You were scolded by a hospital staff member - A hospital staff member made negative comments to you regarding your sexual activity - A hospital staff member threatened that if you did not comply, you or your baby would have a poor outcome (for example you or the baby would be sick or injured)     Q. Did any hospital staff member make negative comments to you about who you are? For example, did you feel you were treated differently based on something about you? (For example, ethnicity, race, religion, level of education, age, marital status?= Yes | All women consented to and completed pre-discharge survey | Any experience of mistreatment  (stratified by type)      Number of women who reported experience of   - Physical abuse (any form) - Verbal abuse (any form) - Stigma and discrimination - Any of the above |
|  |  | **Consent obtained for vaginal examinations.**    - Proportion of women reported that health worker asked for permission before vaginal examination | *Pre-discharge women’s survey:*    Q. Did the health worker ask your permission before performing the vaginal examination? = Yes | All women consented to and completed pre-discharge survey | - Number of women reported that health worker asked for permission before vaginal examination. |
|  |  | **Satisfaction with care**  - Proportion of women who reported satisfaction with care during her hospitalisation for childbirth | Q. Overall, are you satisfied with the care you received during your stay at this hospital for childbirth? Answer = Yes | All women consented to and completed pre-discharge survey | - Number of women who reported being satisfied with care |
|  | Health outcomes | Caesarean section rate (overall, Robson Gs) | *Medical record:*  Q. Mode of birth, parity, plurality, commencement of labour, previous CS, induction, lie of the pregnancy | All women giving birth | Number of women who had   - Any CS - CS by Robson groups |
|  |  | Episiotomy or 3rd/4th degree perineal tears | *Medical record:*  Q. Did the woman receive any of the following interventions or outcomes during labour or birth? = Yes | All women who experienced labour (excludes no labour) | Number of women who had:   - Episiotomy - 3^rd^/4^th^ degree tear |
|  |  | Hysterectomy | *Medical record:*  Q. Did the woman have a hysterectomy prior to discharge? = Yes | All women giving birth | Number of women who had Hysterectomy performed |
| Newborn | Coverage of key practices | **Newborn feeding -** Breastfeeding initiation    - Proportion of women who had breastfeeding started within 1h after birth    - Proportion of women who reported starting breastfeeding in less than 1h after birth | *Medical record:*  Q. When was breastfeeding started? = within 1h after birth | All liveborn babies | Number of newborn who had breastfeeding initiated within 1hour of birth |
|  |  |  | *Pre-discharge women’s survey:*    Q. Howlong after birth did you first start breastfeeding? = less than 1h | All women consented to and completed pre-discharge survey | Number of women who reported starting breastfeeding in less than 1h after birth |
|  | People-centred outcomes | **Skin-to-skin contact**      - Proportion of newborn who had skin-to-skin contact during the first hour after birth                - Proportion of providers who reported encouraging skin-to-skin contact during the hour after birth | *Medical record:*  Q82b – Was skin to skin contact initiated during the first hour after  birth = Yes | All Liveborn babies | Number of newborns who had skin-to-skin contact initiated during the first hour after birth |
|  |  |  | *Pre-discharge women’s survey: (secondary)*    Q. Was your baby placed on your bare chest or abdomen during the hour after birth? = Yes | Women with term singleton births, no major maternal/neonatal complications | Number of women who reported having skin-to-skin contact in the hour after birth |
|  |  |  | *Provider survey:*    Q. Do you encourage skin-to-skin contact between healthy mothers and newborns without complications during the first hour after birth? = Yes (most of the time/always) | All providers providing care during labour and birth | Provider reports encouraging initiating skin to skin contact in the first hour after birth    Number of provider who reported encouraging skin-to-skin contact during the hour after birth |
|  | Health outcomes | **Stillbirth**    - Total stillbirth rate: Number of all stillbirths per 1000 total births    - Antepartum stillbirth rate: Number of antepartum stillbirths per 1000 total births    - Intrapartum stillbirth rate: Number of intrapartum stillbirths per 1000 total births | *Medical record:*    Q. Gestational age at birth    Q. Birth weight    Q. Was the baby alive at birth? = No (antepartum stillbirth/intrapartum stillbirth)    Q. Type of perinatal death = Antepartum/Intrapartum stillbirth | All births ≥28 weeks or >=1000g  Per 1000 total births (liveborn + stillborn) | Number of  - Antepartum stillbirth  - Intrapartum stillbirth    Stillbirth: fetal deaths at ≥28 weeks of gestation, or if gestation is unknown birthweight ≥ 1000 g. |
|  |  | **Early neonatal mortality**    - Early neonatal mortality rate: Number of early neonatal deaths per 1000 live births | Q. Date (and time) of birth of baby    Q. Was the baby alive at birth? = Yes    Q. What was the baby's vital status at hospital discharge, transfer or day 7 after birth (whichever comes first)? = Deceased    Q. Date and time of neonatal discharge/transfer from hospital or death    Q. Type of perinatal death = Early neonatal death | All live births | Number of early neonatal death (0-7day after birth) |
|  |  | **Low birthweight (<2500g)**    - Low birth weight rate | *Medical record:*  Q. birthweight | All births /all livebirths | <2500 grams    Number of newborn weighted <2500g |
|  |  | **Preterm birth** (< 28 weeks, <32 weeks, <37 weeks)      - Preterm birth rate | *Medical record:*  Q. gestational age at birth | All births /all livebirths | Number of newborns with GSA of    <28 weeks  <32 weeks  <37 weeks |
|  |  | **Apgar score less than 7 at 5 minutes**    - Proportion of newborns with Apgar score <7 | *Medical record:*  Q. What was the Apgar score at 5 minutes? | All livebirths | All livebirths with  Apgar score <7    Number of newborn with Apgar score <7 |

| **Individual** | **Type** | **Specific outcome** | **Where measured** | **Denominator** | **Numerator** |
| --- | --- | --- | --- | --- | --- |
| Woman | Coverage of key practices | **Labour monitored using a partograph**    Outcome measures:    - Proportion of women who had their labour monitored using a partograph    - Type and % of each labour monitoring tool used    - Proportion of provider report using a partograph to monitor labour | *Medical record:*  Q. Was the labour monitored using a partograph = Yes    Q. If yes, what type = WHO simplified, WHO LCG, Other | All women who experienced labour (excludes no labour)      Total occurrence (instance) of partograph reported being use | Number of women who had their labour monitored using a partograph    Occurrence (instance) of each partograph reported being use |
|  |  |  | *Provider survey:*  Q25. Do you monitor the progress of a woman’s labour using a partograph or similar tool? = Yes | All providers who provide care during labour and birth | Number of provider reports use of labour monitoring tool (and type of tool) to monitor labour |
|  |  | **Prophylactic uterotonic administered immediately after birth**    Measure:  - Proportion of women who received prophylactic uterotonic immediately after birth | *Medical record:*  Q. Was prophylactic uterotonic given immediately after the birth of the baby? | All women giving birth | Number of women who received uterotonic immediately after birth |
|  |  | **Pre-discharge counselling provided to woman prior to discharge**    Measure:  - Proportion of women who received pre-discharge counselling on each/any/all topics | *Medical record:*    Q. Did the women receive pre-discharge counselling on any of the following topics (Breast feeding, Care of herself, postpartum danger signs and when to seek help, Care of the baby, Newborn danger signs and when to seek help, Family planning method)? = Yes | All women who gave birth and were discharged from hospital alive | Number of women who received pre-discharge counselling on (Each/Any/All) topic |
|  | Women-centred outcomes | **Companion of choice during labour and birth**    Measures:  - Proportion of women who wanted and had a companion during labour      - Proportion of women who wanted and had a companion during birth                        - Proportion of provider who reported that women can have a companion present during labour      - Proportion of provider who reported that women can have a companion present during vaginal birth    - Proportion of provider who reported that women can have a companion present during CS      - Proportion of provider who reported that the health facility has infrastructure to offer companionship | *Pre-discharge women’s survey:*    Q. During labour (before the baby was born), did you want to have a companion in the room to support you (such as a family member or friend)?    Q. Did you have a companion you wanted with you during labour (such as a family member or friend)?    Q. During the birth (when the baby came out), did you want to have a companion in the room to support you (such as a family member or friend)?    Q. Did you have a companion you wanted with you during the birth (such as a family member or friend)? | All women consented to and completed pre-discharge survey     - Who wanted labour companion;      - Who wanted birth companion | - Number of women who had a companion present during labour;    - Number of women who had a companion present during birth |
|  |  |  | *Provider survey: Answer for following Questions = Yes (most of the time/always)*    Q. Are women able to have a companion of their choice (any person chosen by the woman) to provide her with continuous support during labour, for as long as they want?    Q. Are women able to have a companion of their choice (any person chosen by the woman) to provide her with continuous support during vaginal birth, for as long as they want?    Q. Are women able to have a companion of their choice (any person chosen by the woman) to provide her with continuous support during caesarean section, for as long as they want?    Q. This unit has adequate infrastructure, equipment and supplies to offer companionship during labour and childbirth | All providers providing care during labour and birth | Offer women the option of having a companion present during labour/birth      - Number of providers who reported that women are able to have companion during labour    - Number of providers who reported that women are able to have companion during vaginal birth    - Number of providers who reported that women are able to have companion during CS    - Number of providers who reported that the health facility has infrastructure to offer companionship |
|  |  | **Experience of mistreatment**    - Proportion of women reported experiencing physical abuse    - Proportion of women reported experiencing verbal abuse    - Proportion of women reported experiencing stigma and discrimination    - Proportion of women reported any experience of mistreatment | *Pre-discharge women’s survey:*      Q. At any time during your time in hospital, did any of the following occur? = Yes   - You were pinched - You were slapped - Your were physically restrained     Q. During your time in the hospital, did any of the following events occur? = Yes   - You were shouted or screamed at by a hospital staff member - You were scolded by a hospital staff member - A hospital staff member made negative comments to you regarding your sexual activity - A hospital staff member threatened that if you did not comply, you or your baby would have a poor outcome (for example you or the baby would be sick or injured)     Q. Did any hospital staff member make negative comments to you about who you are? For example, did you feel you were treated differently based on something about you? (For example, ethnicity, race, religion, level of education, age, marital status?= Yes | All women consented to and completed pre-discharge survey | Any experience of mistreatment  (stratified by type)      Number of women who reported experience of   - Physical abuse (any form) - Verbal abuse (any form) - Stigma and discrimination - Any of the above |
|  |  | **Consent obtained for vaginal examinations.**    - Proportion of women reported that health worker asked for permission before vaginal examination | *Pre-discharge women’s survey:*    Q. Did the health worker ask your permission before performing the vaginal examination? = Yes | All women consented to and completed pre-discharge survey | - Number of women reported that health worker asked for permission before vaginal examination. |
|  |  | **Satisfaction with care**  - Proportion of women who reported satisfaction with care during her hospitalisation for childbirth | Q. Overall, are you satisfied with the care you received during your stay at this hospital for childbirth? Answer = Yes | All women consented to and completed pre-discharge survey | - Number of women who reported being satisfied with care |
|  | Health outcomes | Caesarean section rate (overall, Robson Gs) | *Medical record:*  Q. Mode of birth, parity, plurality, commencement of labour, previous CS, induction, lie of the pregnancy | All women giving birth | Number of women who had   - Any CS - CS by Robson groups |
|  |  | Episiotomy or 3rd/4th degree perineal tears | *Medical record:*  Q. Did the woman receive any of the following interventions or outcomes during labour or birth? = Yes | All women who experienced labour (excludes no labour) | Number of women who had:   - Episiotomy - 3^rd^/4^th^ degree tear |
|  |  | Hysterectomy | *Medical record:*  Q. Did the woman have a hysterectomy prior to discharge? = Yes | All women giving birth | Number of women who had Hysterectomy performed |
| Newborn | Coverage of key practices | **Newborn feeding -** Breastfeeding initiation    - Proportion of women who had breastfeeding started within 1h after birth    - Proportion of women who reported starting breastfeeding in less than 1h after birth | *Medical record:*  Q. When was breastfeeding started? = within 1h after birth | All liveborn babies | Number of newborn who had breastfeeding initiated within 1hour of birth |
|  |  |  | *Pre-discharge women’s survey:*    Q. Howlong after birth did you first start breastfeeding? = less than 1h | All women consented to and completed pre-discharge survey | Number of women who reported starting breastfeeding in less than 1h after birth |
|  | People-centred outcomes | **Skin-to-skin contact**      - Proportion of newborn who had skin-to-skin contact during the first hour after birth                - Proportion of providers who reported encouraging skin-to-skin contact during the hour after birth | *Medical record:*  Q82b – Was skin to skin contact initiated during the first hour after  birth = Yes | All Liveborn babies | Number of newborns who had skin-to-skin contact initiated during the first hour after birth |
|  |  |  | *Pre-discharge women’s survey: (secondary)*    Q. Was your baby placed on your bare chest or abdomen during the hour after birth? = Yes | Women with term singleton births, no major maternal/neonatal complications | Number of women who reported having skin-to-skin contact in the hour after birth |
|  |  |  | *Provider survey:*    Q. Do you encourage skin-to-skin contact between healthy mothers and newborns without complications during the first hour after birth? = Yes (most of the time/always) | All providers providing care during labour and birth | Provider reports encouraging initiating skin to skin contact in the first hour after birth    Number of provider who reported encouraging skin-to-skin contact during the hour after birth |
|  | Health outcomes | **Stillbirth**    - Total stillbirth rate: Number of all stillbirths per 1000 total births    - Antepartum stillbirth rate: Number of antepartum stillbirths per 1000 total births    - Intrapartum stillbirth rate: Number of intrapartum stillbirths per 1000 total births | *Medical record:*    Q. Gestational age at birth    Q. Birth weight  Q. Was the baby alive at birth? = No (antepartum stillbirth/intrapartum stillbirth)    Q. Type of perinatal death = Antepartum/Intrapartum stillbirth | All births ≥28 weeks or >=1000g  Per 1000 total births (liveborn + stillborn) | Number of  - Antepartum stillbirth  - Intrapartum stillbirth    Stillbirth: fetal deaths at ≥28 weeks of gestation, or if gestation is unknown birthweight ≥ 1000 g. |
|  |  | **Early neonatal mortality**    - Early neonatal mortality rate: Number of early neonatal deaths per 1000 live births | Q. Date (and time) of birth of baby    Q. Was the baby alive at birth? = Yes    Q. What was the baby's vital status at hospital discharge, transfer or day 7 after birth (whichever comes first)? = Deceased    Q. Date and time of neonatal discharge/transfer from hospital or death    Q. Type of perinatal death = Early neonatal death | All live births | Number of early neonatal death (0-7day after birth) |
|  |  | **Low birthweight (<2500g)**    - Low birth weight rate | *Medical record:*  Q. birthweight | All births /all livebirths | <2500 grams    Number of newborn weighted <2500g |
|  |  | **Preterm birth** (< 28 weeks, <32 weeks, <37 weeks)  - Preterm birth rate | *Medical record:*  Q. gestational age at birth | All births /all livebirths | Number of newborns with GSA of  <28 weeks  <32 weeks  <37 weeks |
|  |  | **Apgar score less than 7 at 5 minutes**    - Proportion of newborns with Apgar score <7 | *Medical record:*  Q. What was the Apgar score at 5 minutes? | All livebirths | All livebirths with  Apgar score <7    Number of newborn with Apgar score <7 |
